# Supplementary material for: Inhibitory Concentrations of Ciprofloxacin Induce an Adaptive Response Promoting the Intracellular Survival of Salmonella enterica Serovar Typhimurium
Source: mBio. 2021 Jun 22;12(3):e01093-21. doi: 10.1128/mBio.01093-21 (PMC8262899; doi:10.1128/mBio.01093-21)
Supplement: FIG S1 [file mbio.01093-21-sf001.docx]

**Figure S1. Time kill curves of *S.* Typhimurium under ciprofloxacin exposure to assess ciprofloxacin stability. A.** Time kill curves were performed on *S.* Typhimurium D23580 using spent medium following an initial 24-hour kill curve. Media for this growth curve was centrifuged and steri-filtered before inoculation with D23580 and growth over 24 hours. CFU were enumerated at 6 time points, and two independent biological replicates were plotted. **B.** Average CFU/ml were plotted as mean ± SD for the 24 hour time point to compare CFU between treatment conditions. An ANOVA was performed to compare means at 24 hours, and Dunnett’s test was performed to compare 24 hour means of 1x, 2x, and 4x ciprofloxacin MIC to 0x (control).
