# Supplementary material for: Inhibitory Concentrations of Ciprofloxacin Induce an Adaptive Response Promoting the Intracellular Survival of Salmonella enterica Serovar Typhimurium
Source: mBio. 2021 Jun 22;12(3):e01093-21. doi: 10.1128/mBio.01093-21 (PMC8262899; doi:10.1128/mBio.01093-21)
Supplement: TABLE S1 [file mbio.01093-21-st001.docx]

**Table S1**. **Top 20 significantly upregulated genes in 2x MIC ciprofloxacin-treated D23580 relative to NT.**

| Gene name | Higher function | Function | Log_2_ fold change | Adjusted p-value |
| --- | --- | --- | --- | --- |
| *abc2* | Phage | BTP1 anti-RecBCD | 7.90 | 2.11E-158 |
| STMMW_03422 |  | BTP1 predicted prophage protein | 7.90 | 9.40E-158 |
| *arf* |  | BTP1 predicted prophage protein | 7.80 | 8.85E-117 |
| STMMW_32081 |  | exonuclease | 7.68 | 4.69E-35 |
| STMMW_32082 |  | predicted phage protein | 7.66 | 1.48E-31 |
| *EaE* |  | BTP1 predicted prophage protein | 7.65 | 1.44E-142 |
| STMMW_32091 |  | predicted phage protein | 7.63 | 4.18E-31 |
| *kil* |  | BTP1 kil | 7.58 | 6.48E-104 |
| *erf* |  | BTP1 predicted prophage protein | 7.51 | 2.37E-144 |
| STMMW_32071 |  | predicted phage protein | 7.48 | 8.41E-36 |
| *abc1* |  | BTP1 anti-RecBCD | 7.47 | 2.99E-134 |
| *cIII* |  | BTP1 regulatory protein cIII | 7.35 | 1.17E-98 |
| STMMW_32101 |  | predicted phage protein | 7.35 | 1.06E-23 |
| STMMW_32061 |  | predicted phage protein | 7.34 | 5.23E-40 |
| *EaF* |  | BTP1 predicted prophage protein | 7.28 | 2.42E-192 |
| STMMW_03801 |  | BTP1 scaffolding protein | 6.88 | 2.23E-87 |
| STMMW_03491 |  | BTP1 predicted prophage protein | 6.89 | 2.23E-87 |
| STMMW_32112 |  | phage regulatory protein | 6.87 | 4.22E-19 |
| *ysdB* (*tisB*) | DNA-damage, SOS response | putative LexA-regulated protein TisB | 7.35 | 7.06E-138 |
| *ysdA* (*tisA*) | (Toxin-antitoxin system) |  | 7.34 | 9.36E-151 |
