# Supplementary material for: Inhibitory Concentrations of Ciprofloxacin Induce an Adaptive Response Promoting the Intracellular Survival of Salmonella enterica Serovar Typhimurium
Source: mBio. 2021 Jun 22;12(3):e01093-21. doi: 10.1128/mBio.01093-21 (PMC8262899; doi:10.1128/mBio.01093-21)
Supplement: TABLE S2 [file mbio.01093-21-st002.docx]

**Table S2**. **Top 20 significantly downregulated genes in 2x MIC ciprofloxacin-treated D23580 relative to NT.**

| Gene name | Higher function | Function | Log_2_ fold change | Adjusted p-value |
| --- | --- | --- | --- | --- |
| *fimC* | Pili-formation, host cell attachment | fimbrial chaperone protein | -3.50 | 1.68E-37 |
| *ymdA* |  | putative exported protein | -3.28 | 3.25E-53 |
| *fimI* |  | major pilin protein | -2.71 | 8.85E-55 |
| *flgH* | Flagella formation | Basal body L-ring protein | -3.13 | 1.25E-124 |
| *flgI* |  | Basal body P-ring protein | -3.10 | 8.43E-84 |
| *flgJ* |  | flagellar protein FlgJ | -3.01 | 1.65E-76 |
| *flgG* |  | flagellar basal-body rod protein FlgG (distal rod protein) | -2.85 | 4.11E-59 |
| *flgB* |  | putative flagellar basal-body rod protein FlgB (proximal rod protein) | -2.84 | 4.41E-67 |
| *flgD* |  | flagellar hook formation protein FlgD | -2.82 | 5.86E-76 |
| *flgC* |  | putative flagellar basal-body rod protein FlgC (proximal rod protein) | -2.81 | 3.59E-66 |
| *flgF* |  | putative flagellar basal-body rod protein FlgF (proximal rod protein) | -2.81 | 2.51E-152 |
| *flgE* |  | flagellar hook protein FlgE | -2.81 | 1.36E-129 |
| *fliJ* |  | flagellar FliJ protein | -2.64 | 1.39E-174 |
| *flhE* |  | flagellar protein FlhE precursor | -2.63 | 7.58E-42 |
| *fliR* |  | flagellar biosynthetic protein FliR | -2.63 | 2.63E-44 |
| *ompD* | Porin | outer membrane porin protein (ompD) | -2.90 | 7.08E-42 |
| *srfA* | SPI-1 associated, virulence | putative virulence effector protein | -2.82 | 1.04E-73 |
| *srfB* |  | putative virulence effector protein | -2.76 | 0 |
| STMMW_19161 | Metabolism | putative lipoprotein | -2.63 | 6.05E-23 |
| *tnpA_2b* | DNA recombination, transposition | transposase for insertion sequence element IS200 | -2.61 | 5.49E-25 |
