# Supplementary material for: Inhibitory Concentrations of Ciprofloxacin Induce an Adaptive Response Promoting the Intracellular Survival of Salmonella enterica Serovar Typhimurium
Source: mBio. 2021 Jun 22;12(3):e01093-21. doi: 10.1128/mBio.01093-21 (PMC8262899; doi:10.1128/mBio.01093-21)
Supplement: TABLE S3 [file mbio.01093-21-st003.docx]

**Table S3. Top 20 significantly upregulated genes in 1x MIC azithromycin D23580 relative to NT.**

| Gene name | Higher function | Function | Log_2_ fold change | Adjusted *p*-value |
| --- | --- | --- | --- | --- |
| *ycfR* | **Membrane-associated** | putative secreted protein | 4.34 | 2.68E-104 |
| STMMW_01051 |  | putative inner membrane protein | 2.32 | 0.005 |
| *ilvG* | **Metabolism** | acetolactate synthase large subunit | 3.37 | 4.67E-208 |
| *finP* |  | putative transglycosylase | 2.93 | 4.70E-100 |
| STMMW_27191 |  | glucitol/sorbitol-specific phosphotransferase enzyme IIB component | 2.86 | 2.81E-23 |
| STMMW_27181 |  | putative PTS enzyme III glucitol | 2.65 | 3.68E-21 |
| *ilvM* |  | acetohydroxy acid synthase II, small subunit | 2.44 | 1.99E-71 |
| *cdsH* |  | putative lyase | 2.21 | 7.83E-59 |
| *sseA* | **SPI-2, virulence** | putative pathogenicity island protein | 2.30 | 3.83E-63 |
| STMMW_22601 |  | putative SsrB-regulated factor | 2.27 | 1.67E-08 |
| *ssaR* |  | putative type III secretion protein | 2.24 | 1.89E-87 |
| *nrdI* | **Redox, electron transport chain** | NrdI protein | 2.23 | 6.34E-16 |
| STMMW_10361 | **Phage** | predicted prophage protein | 2.28 | 7.13E-154 |
| SLT-BT0451 | **Others** | transposase (pseudogene) | 2.83 | 2.77E-78 |
| STMMW_01681 |  | conserved hypothetical protein | 2.61 | 1.51E-90 |
| STMMW_01041 |  | - | 2.60 | 0.022 |
| STMMW_03081 |  | putative IS1400 transposase B (pseudogene) | 2.56 | 0.012 |
| *ybfA* |  | putative exported protein | 2.55 | 6.83E-26 |
| *tniB* |  | IS-element ATP-binding protein (pseudogene) | 2.42 | 8.27E-46 |
| STMMW_03251 |  | release factor H-coupled RctB family protein (pseudogene) | 2.29 | 6.43E-73 |
