# Supplementary material for: Inhibitory Concentrations of Ciprofloxacin Induce an Adaptive Response Promoting the Intracellular Survival of Salmonella enterica Serovar Typhimurium
Source: mBio. 2021 Jun 22;12(3):e01093-21. doi: 10.1128/mBio.01093-21 (PMC8262899; doi:10.1128/mBio.01093-21)
Supplement: TABLE S4 [file mbio.01093-21-st004.docx]

**Table S4. Top 20 significantly downregulated genes in 1x MIC azithromycin D23580 relative to NT.**

| Gene name | Higher function | Function | Log_2_ fold change | Adjusted *p*-value |
| --- | --- | --- | --- | --- |
| *flgD* | **Motility** | flagellar hook formation protein FlgD | -6.57 | 0 |
| *flgC* |  | putative flagellar basal-body rod protein FlgC (proximal rod protein) | -6.43 | 5.37E-267 |
| *flgE* |  | flagellar hook protein FlgE | -6.08 | 0 |
| *flgB* |  | putative flagellar basal-body rod protein FlgB (proximal rod protein) | -6.00 | 1.50E-247 |
| *flgG* |  | flagellar basal-body rod protein FlgG (distal rod protein) | -5.80 | 2.56E-219 |
| *fliH* |  | flagellar assembly protein FliH | -5.35 | 0 |
| *fliJ* |  | flagellar FliJ protein | -5.34 | 0 |
| *fliN* |  | flagellar motor switch protein FliN | -5.20 | 0 |
| *flgH* |  | Basal body L-ring protein | -5.15 | 1.53E-295 |
| *fliI* |  | flagellum-specific ATP synthase | -5.09 | 0 |
| *fliG* |  | flagellar motor switch protein FliG | -5.09 | 0 |
| *flgI* |  | Basal body P-ring protein | -4.97 | 8.46E-200 |
| *flgF* |  | putative flagellar basal-body rod protein FlgF (proximal rod protein) | -4.85 | 0 |
| *fliM* |  | flagellar motor switch protein FliM | -4.84 | 0 |
| *fliA* |  | RNA polymerase sigma transcription factor for flagellar operon | -4.76 | 2.15E-129 |
| *flgJ* |  | flagellar protein FlgJ | -4.52 | 2.39E-163 |
| *fliF* |  | flagellar basal-body M-ring protein | -4.52 | 1.18E-232 |
| *fliO* |  | flagellar protein FliO | -4.49 | 0 |
| *fliE* |  | flagellar hook-basal body complex protein FliE | -4.36 | 2.18E-49 |
| STMMW_39661 | **Other** | hypothetical protein | -4.40 | 1.96E-55 |
