# Supplementary material for: Inhibitory Concentrations of Ciprofloxacin Induce an Adaptive Response Promoting the Intracellular Survival of Salmonella enterica Serovar Typhimurium
Source: mBio. 2021 Jun 22;12(3):e01093-21. doi: 10.1128/mBio.01093-21 (PMC8262899; doi:10.1128/mBio.01093-21)
Supplement: TABLE S5 [file mbio.01093-21-st005.docx]

**Table S5. Top 20 significantly upregulated genes in 50% sucrose fraction of ciprofloxacin-treated D23580 relative to NT.**

| **Gene name** | **Higher function** | **Function** | **Log_2_ fold change** | **Adjusted *p*-value** |
| --- | --- | --- | --- | --- |
| STMMW_32091 | **Phage** | predicted phage protein | 8.27 | 1.05E-136 |
| STMMW_32082 |  | predicted phage protein | 8.26 | 3.47E-162 |
| STMMW_32081 |  | exonuclease | 8.17 | 4.15E-193 |
| STMMW_32071 |  | predicted phage protein | 8.05 | 4.47E-195 |
| STMMW_32112 |  | phage regulatory protein | 7.91 | 8.45E-70 |
| STMMW_32061 |  | predicted phage protein | 7.78 | 8.08E-206 |
| STMMW_32101 |  | predicted phage protein | 7.75 | 4.92E-108 |
| *cIIb* |  | phage regulatory protein | 7.72 | 8.91E-65 |
| *erf* |  | BTP1 predicted prophage protein | 7.50 | 0 |
| STMMW_20261 |  | predicted phage protein | 7.48 | 4.07E-244 |
| STMMW_20251 |  | predicted phage protein | 7.47 | 5.57E-245 |
| *arf* |  | BTP1 predicted prophage protein | 7.46 | 8.19E-202 |
| *abc2* |  | BTP1 anti-RecBCD | 7.36 | 1.17E-151 |
| STMMW_20271 |  | predicted phage protein | 7.24 | 6.99E-106 |
| STMMW_03422 |  | BTP1 predicted prophage protein | 7.20 | 2.18E-250 |
| *kil* |  | BTP1 kil | 7.15 | 1.96E-210 |
| *abc1* |  | BTP1 anti-RecBCD | 7.14 | 2.18E-187 |
| STMMW_20281 |  | predicted phage protein | 7.08 | 1.11E-84 |
| *ysdA* (*tisA*) | **DNA-damage, SOS response** | conserved hypothetical protein | 7.92 | 6.06E-199 |
| *ysdB (tisB)* | **(Toxin-antitoxin system)** | putative LexA-regulated protein TisB | 7.73 | 1.28E-222 |
