# Supplementary material for: Inhibitory Concentrations of Ciprofloxacin Induce an Adaptive Response Promoting the Intracellular Survival of Salmonella enterica Serovar Typhimurium
Source: mBio. 2021 Jun 22;12(3):e01093-21. doi: 10.1128/mBio.01093-21 (PMC8262899; doi:10.1128/mBio.01093-21)
Supplement: TABLE S6 [file mbio.01093-21-st006.docx]

**Table S6. 20 top downregulated genes in 50% sucrose fraction of ciprofloxacin-treated D23580 relative to NT.**

| Gene name | Higher function | Function | Log_2_ fold change | Adjusted *p*-value |
| --- | --- | --- | --- | --- |
| *citC* | **Metabolism** | citrate (PRO-3S)-lyase ligase | -3.94 | 6.22E-30 |
| *citD* |  | citrate lyase acyl carrier protein | -3.78 | 9.25E-25 |
| *citE* |  | citrate lyase beta chain | -3.51 | 3.29E-21 |
| *narH* |  | respiratory nitrate reductase 1 beta chain | -3.42 | 4.52E-46 |
| *citF* |  | citrate lyase alpha chain | -3.41 | 6.51E-14 |
| *prpR* |  | propionate catabolism operon regulatory protein | -3.36 | 6.51E-08 |
| *narG* |  | respiratory nitrate reductase 1 alpha chain | -3.25 | 3.29E-26 |
| *narJ* |  | respiratory nitrate reductase 1 delta chain | -3.08 | 2.12E-33 |
| *asnB* |  | asparagine synthetase B | -3.07 | 2.26E-09 |
|  |  |  |  |  |
| *cstAb* (*yjiY*) |  | probable carbon starvation protein | -3.21 | 3.25E-37 |
| *glpB* |  | Anaerobic G-3-P dehydrogenase subunit B | -2.87 | 5.19E-06 |
| *ccmB* | **Redox, electron transport chain** | Heme exporter protein B | -3.69 | 0.002 |
| *dmsA* |  | anaerobic dimethyl sulfoxide reductase chain A precursor | -3.17 | 1.11E-16 |
| *dmsB* |  | anaerobic dimethyl sulfoxide reductase chain B | -3.11 | 3.72E-15 |
| *napG* |  | quinole dehydrogenase periplasmic component | -2.95 | 1.31E-19 |
| STMMW_12381 | **DNA recombination** | putative transposase | -3.40 | 1.62E-12 |
| *ymdA* | **Biofilm formation** | putative exported protein | -2.99 | 1.40E-45 |
| *yddX* | **Others** | conserved hypothetical protein | -3.51 | 2.72E-83 |
| *hdeB* |  | putative secreted acid resistance protein | -3.16 | 9.06E-29 |
| *hscC* |  | chaperone heat shock protein | -3.01 | 1.20E-20 |
