# Supplementary material for: Inhibitory Concentrations of Ciprofloxacin Induce an Adaptive Response Promoting the Intracellular Survival of Salmonella enterica Serovar Typhimurium
Source: mBio. 2021 Jun 22;12(3):e01093-21. doi: 10.1128/mBio.01093-21 (PMC8262899; doi:10.1128/mBio.01093-21)
Supplement: TABLE S7 [file mbio.01093-21-st007.docx]

**Table S7. Top 20 significantly upregulated genes in ciprofloxacin-treated D23580 60% sucrose fraction relative to ciprofloxacin-treated D23580 50% fraction.**

| Gene name | Higher function | Function | Log_2_ fold change | Adjusted *p*-value |
| --- | --- | --- | --- | --- |
| STMMW_31971 | **Phage** | putative major capsid protein | 1.49 | 0.03 |
| STMMW_31951 |  | terminase, endonuclease subunit | 1.48 | 0.02 |
| STMMW_03602 |  | BTP1 predicted prophage protein | 1.42 | 3.63E-13 |
| STMMW_31941 |  | putative capsid completion protein | 1.41 | 0.03 |
| STMMW_03601 |  | BTP1 predicted prophage protein | 1.41 | 1.71E-09 |
| *ninF* |  | BTP1 predicted prophage protein | 1.29 | 4.78E-08 |
| STMMW_03821 |  | BTP1 predicted prophage protein | 1.27 | 0.0003 |
| *ninE* |  | BTP1 predicted prophage protein | 1.25 | 1.56E-09 |
| STMMW_03611 |  | BTP1 predicted prophage protein | 1.24 | 9.66E-14 |
| *ninB* |  | BTP1 predicted prophage protein | 1.21 | 7.66E-15 |
| STMMW_32101 |  | predicted phage protein | 1.19 | 0.005 |
| STMMW_03621 |  | BTP1 predicted prophage protein | 1.12 | 4.43E-15 |
| STMMW_03811 |  | BTP1 coat protein | 1.11 | 1.31E-06 |
| STMMW_03801 |  | BTP1 scaffolding protein | 1.11 | 6.42E-06 |
| *ninG* |  | BTP1 predicted prophage protein | 1.11 | 3.94E-27 |
| STMMW_03771 |  | BTP1 terminase small subunit | 1.07 | 8.35E-09 |
| STMMW_03582 |  | BTP1 predicted prophage protein | 1.07 | 1.18E-21 |
| STMMW_03861 |  | BTP1 head assembly protein | 1.06 | 0.003 |
| STMMW_03881 |  | BTP1 DNA transfer protein | 1.06 | 0.0007 |
| STMMW_03791 |  | BTP1 portal protein | 1.05 | 2.45E-07 |
