# Supplementary material for: Inhibitory Concentrations of Ciprofloxacin Induce an Adaptive Response Promoting the Intracellular Survival of Salmonella enterica Serovar Typhimurium
Source: mBio. 2021 Jun 22;12(3):e01093-21. doi: 10.1128/mBio.01093-21 (PMC8262899; doi:10.1128/mBio.01093-21)
Supplement: TABLE S8 [file mbio.01093-21-st008.docx]

**Table S8. Top 20 significantly downregulated genes in ciprofloxacin-treated D23580 60% sucrose fraction relative to ciprofloxacin-treated D23580 50% fraction.**

| Gene name | Higher function | Function | Log_2_ fold change | Adjusted *p*-value |
| --- | --- | --- | --- | --- |
| *invF* | **SPI-1, virulence** | possible AraC-family regulatory protein | -4.88 | 9.06E-20 |
| *invG* |  | secretory protein (associated with virulence) | -4.74 | 1.57E-19 |
| *spaQ* |  | secretory protein (associated with virulence) | -4.72 | 4.97E-22 |
| *invE* |  | cell invasion protein | -4.60 | 1.47E-16 |
| *spaP* |  | secretory protein (associated with virulence) | -4.51 | 1.67E-25 |
| *invA* |  | possible secretory protein (associated with virulence) | -4.39 | 1.19E-20 |
| *spaR* |  | secretory protein (associated with virulence) | -4.28 | 6.65E-18 |
| *invJ* |  | surface presentation of antigens protein (associated with type III secretion and virulence) | -4.24 | 1.36E-21 |
| *invC* |  | secretory apparatus ATP synthase (associated with virulence) | -4.22 | 2.27E-23 |
| *sipD* |  | pathogenicity island 1 effector protein | -4.19 | 1.36E-12 |
| *sipB* |  | pathogenicity island 1 effector protein | -4.19 | 2.92E-15 |
| *sicA* (*spaT*) |  | unknown function | -4.17 | 1.45E-16 |
| *invI* (*spaM*) |  | secretory protein (associated with virulence) | -4.17 | 8.33E-13 |
| *sipC* |  | pathogenicity island 1 effector protein | -4.15 | 2.16E-17 |
| *spaO* |  | surface presentation of antigens protein (associated with type III secretion and virulence) | -4.15 | 3.44E-23 |
| *spaS* |  | secretory protein (associated with virulence) | -4.10 | 2.30E-19 |
| *sipA* |  | pathogenicity island 1 effector protein | -4.02 | 9.43E-13 |
| *ssaI* | **SPI-2, virulence** | putative pathogenicity island protein | -4.22 | 3.89E-12 |
| *ssaG* |  | putative pathogenicity island protein | -4.21 | 3.96E-11 |
| *ssaC* (*spiA*) |  | putative outer membrane secretory protein | -4.03 | 7.09E-18 |
